# Assessment of Protective Effects of Carvacrol on Haloperidol-Induced Oxidative Stress and Genotoxicity in Human Peripheral Blood Lymphocytes

**DOI:** 10.1155/2022/9565881

**Published:** 2022-10-25

**Authors:** Ehsan Zamani, Alireza Ahmadi Shad, Hediye Fatemi, Saba Mahboubi, Azadeh Motavallian, Mehdi Evazalipour

**Affiliations:** ^1^Department of Pharmacology and Toxicology, School of Pharmacy, Guilan University of Medical Sciences, Rasht, Iran; ^2^Student Research Committee, School of Pharmacy, Guilan University of Medical Sciences, Rasht, Iran; ^3^Department of Pharmaceutical Biotechnology, School of Pharmacy, Guilan University of Medical Sciences, Rasht, Iran; ^4^Department of Gastroenterology and Hepatology, Erasmus University Medical Center, Rotterdam, Netherlands

## Abstract

Haloperidol is a first-generation antipsychotic drug that has several indications in a wide range of mental conditions. The extensive prescription of haloperidol is correlated with some less-known adverse effects such as genotoxicity. Carvacrol is a monoterpenoid mainly found in oregano and thyme. It has the potential to scavenge free radicals in addition to increasing antioxidant defense enzyme activities and glutathione levels. In this study, we attempted to explore the possible potential of haloperidol in inducing genotoxicity in human peripheral lymphocytes as well as the protective role of carvacrol against this effect. The lymphocytes were divided into separate groups as follows: control group (cosolvent and NS); carvacrol group (5 *μ*M); haloperidol group (25, 50, and 100 ng/ml); haloperidol (25, 50, and 100 ng/ml) + carvacrol (5 *μ*M); positive control (0.8 *μ*g/ml Cisplatin). After 24 hours of treatment, we conducted a cytokinesis-Block micronucleus test and an alkaline comet assay in order to determine genetic damage. Additionally, we measured glutathione and MDA levels as the biomarkers associated with oxidative stress. Significant increases in the levels of genotoxicity biomarkers (micronucleus frequency, DNA percentage in tail and tail moment) were observed in haloperidol-treated cells. The result of our oxidative stress tests also demonstrated that haloperidol had the potential to induce oxidative stress via reducing the levels of glutathione and increasing lipid peroxidation. Treatment with carvacrol significantly decreased the genotoxic events. It can be presumed that the induction of oxidative stress by haloperidol is the critical event associated with haloperidol-mediated genotoxicity. Therefore, using carvacrol as a natural antioxidant protected human lymphocytes against haloperidol genetic damage.

## 1. Introduction

Antipsychotic drugs are commonly prescribed to manage the symptoms of various psychiatric conditions, in particular schizophrenia and bipolar disorder [1]. Generally, they are classified into first generation and second-generation antipsychotics [2]. It is assumed that this class of drugs mainly acts by blocking dopaminergic signal transmission in the nigrostriatal pathway. Due to the aforementioned mechanism of action, it is of high probability that the side effects associated with the motor system, including extrapyramidal syndrome, may be observed [3, 4].

Haloperidol is classified as a typical antipsychotic drug and is extensively prescribed in order to manage various acute and chronic psychiatric conditions, such as schizophrenia, Tourette syndrome, different behavioral disorders in children, and acute mania. Also, haloperidol can be prescribed for chemotherapy-induced nausea and vomiting, and persistent hiccups [5–12]. Like any other first-generation antipsychotic, haloperidol mainly acts by blocking dopaminergic receptors, in particular D2 receptors. The blockade of the dopaminergic pathway consequently leads to numerous side effects, such as tardive dyskinesia [13]. Chronic administration of haloperidol is assumed to be associated with an increase in the turnover rate of dopamine, which can possibly result in the production of reactive oxygen species (ROS) [14]. Moreover, it is proposed that haloperidol may cause a remarkable decline in the amount of glutathione [15].

The excessive production of free radicals, such as ROS, and the inability of the antioxidant defense system to stabilize them leads to a situation referred to as oxidative stress [16]. The probable role of free radicals in damaging macromolecules, like DNAs, proteins, and lipids, has been demonstrated in several studies [17–20]. More specifically, the results of some studies have illustrated the harmful effects of excessive ROS production on the genetic components of cells [21–25].

There have been promising reports on the major role of natural antioxidants in ameliorating oxidative stress [26, 27]. Carvacrol, (2-methyl-5-(1-methylethyl)-phenol), is a phenolic monoterpenoid, which can be found predominantly in the essential oils of various plants, such as oregano and thyme [28]. It has numerous beneficial features, including antibacterial, antifungal, antiviral, and antiproliferative activities [29–32]. Additionally, potential antioxidant properties have been observed with carvacrol. It is assumed that one of the main mechanisms is the inhibition of lipid peroxidation [33, 34].

As regards the extensive use of haloperidol, it is of high significance to evaluate the toxic effects, which are more obscure. Furthermore, there are few studies on haloperidol's probable genotoxicity. Therefore, the main objective of this study is to assess the possible genotoxic activity of haloperidol as well as the protective effects of carvacrol on human peripheral blood lymphocyte cells.

## 2. Materials and Methods

### 2.1. Chemicals

Haloperidol, carvacrol, phytohaemagglutinin (PHA), cytochalasin B, thiobarbituric acid, Tris-HCl, Tris ammonium, MgCl_2,_ Disodium hydrogen phosphate, TCA, sucrose, EDTA, sodium acetate, Triton X-100, and Giemsa stain were from Sigma; DMEM cell culture and phosphate buffered saline (PBS) were from Gibco. Phosphoric acid, methanol, acetic acid glacial, potassium chloride, n-Butanol, sodium chloride, DTNB, Na_2_EDTA, sodium hydroxide, sodium lauroyl sarcosinate, DMSO, and normal melting point agarose were from Merck. The low melting point (LMP) agarose was from Cleaver Scientific.

### 2.2. Blood Sampling and Treatment

The heparinized blood sample was obtained from the donor (a healthy, young, nonalcoholic, nonsmoking male). The donor had not been exposed to any chemicals or ionizing radiation that might have interfered with the results of the test throughout the six-month period of time prior to the blood sampling. An informed consent form was signed by the donor. The study was approved by the Guilan University of Medical Sciences' Ethics Committee.

The blood sample was mixed with DMEM, which also contained 10% FBS, Glutamine, and Pen/Strep. The resulting mixture was divided into separate groups as follows:

  Control group (cosolvent and NS);
  Carvacrol group (5 *μ*M) [35];
  Haloperidol group (25, 50, and 100 ng/ml) [36];
  Haloperidol (25, 50, and 100 ng/ml) + carvacrol (5 *μ*M);
  Positive control (0.8 *μ*g/ml Cisplatin).

After that, all groups were incubated for 24 hours (under the conditions of 37°C and 5% CO_2_ pressure).

### 2.3. Cytokinesis-Block Micronucleus Assay (CBMN Assay)

The cytokinesis-block micronucleus assay (CBMN assay) was conducted as previously described with minor modifications [16]. Phytohaemagglutinin (PHA) was added to all the mentioned groups in order to stimulate mitosis in the culture. Subsequently, the cell mixtures were incubated for 72 hours. After 44 hours, cytochalasin-B (3 *μ*g/ml) was added to the samples, leading to the inhibition of cytokinesis. After 28 hours, the culture was harvested, all groups were centrifuged at 200 × *g* and the supernatants were discarded. Subsequently, the samples were treated with hypotonic KCl followed by the addition of a prefixing solution (3 : 5, methanol: acetic acid) to the pellet. Right after that, the samples were centrifuged at 146 × *g* for 10 minutes, discarding the supernatant. Then, a fixative solution (3 : 1, methanol: acetic acid) was added. This stage was carried out two times. Next, a few drops of cell mixture were dropped on slides. Following that, the samples were air-dried. Eventually, the samples were stained using a 5% Giemsa solution. At least one thousand binuclear lymphocytes were analyzed under a microscope (Micros Austria daffodil MCX100, Vienna, Austria) at 400X magnification to determine the frequency of micronuclei (MN).

### 2.4. Single-Cell Gel Electrophoresis Assay (Alkaline Comet Assay)

The alkaline comet assay was conducted as stated by Singh et al. with minor modifications [37]. The samples were centrifuged at 200 × *g* and the pellet pertaining to each sample group was mixed with 1% LMP agarose. The resultant mixtures were layered on fully frosted slides, which were already precoated with 1% normal melting point agarose. Immediately after that, the slides were covered and placed in a dark place for 10 minutes at 4°C in order for the LMP agarose to solidify. Subsequently, the samples were placed in a lysis solution at 4°C and in a dark chamber for 24 hours. Following lysis, the samples were placed horizontally in an alkaline electrophoresis buffer for 20 minutes at 4°C, and right after that, the electrophoresis was conducted for 20 minutes at 4°C, 1 V/cm, and 300 mA. Eventually, the slides were rinsed using Tris buffer as a neutralizing solution and then dehydrated with ethanol. This stage was repeated two more times. Next, the slides were stained using SYBR® Gold stain for 15 minutes in the dark and rinsed with deionized water in order to remove excess stain. A total number of 100 nucleotides per slide were analyzed using a fluorescence invert microscope (Nikon Eclipse TS100, Tokyo, Japan) at 200X magnification. In order to quantify DNA damage, the head and tail intensities were measured using CASPLab® (CASP1.2.3 beta2) software, and the tail moment was calculated afterwards.

### 2.5. Measurement of Oxidative Stress Parameters

#### 2.5.1. Measurement of Lipid Peroxidation

Thiobarbituric acid was used in order to measure malondialdehyde (MDA) levels as previously claimed with minor modifications [38]. After that, 0.05 M sulfuric acid was added to the cell homogenates, and after that, 0.2% TBA was added to the cell mixtures. Following that, for 30 minutes, the samples were put in a boiling water bath. Right after that, the samples were placed on ice, and n-butanol was added to all groups at the same time. Subsequently, the cell mixtures were centrifuged at 3500 × *g* for 10 minutes. Eventually, the supernatant of each group was analyzed with three replicates at 532 nm using an ELISA microplate reader. The malondialdehyde level was determined from a standard curve and the malondialdehyde level was expressed as *µ*M.

#### 2.5.2. Measurement of Glutathione Content

Glutathione content was assessed as it was previously described, with minor modifications [38]. DTNB (5, 5-dithio-bis-(2-nitrobenzoic acid) was used as the indicator for the determination of glutathione concentration. Then, TCA was added to the cell suspensions in order to precipitate the proteins, and then the samples were centrifuged at 5000 × *g* for 5 minutes at 4°C. Next, the supernatant pertaining to each group was mixed with DTNB and PBS. The samples were evaluated at 412 nm using a spectrophotometer. A standard curve was used to determine total glutathione . The ottal glutathione concentration was expressed as *µ*M glutathione.

### 2.6. Statistical Analysis

All statistical analyses were performed using GraphPad Prism® software (version 6). The results were expressed as the mean ± SD. The assays were performed in at least triplicate. Comparison between groups was made using the one-way ANOVA test, followed by the post hoc Tukey's test. *P* < 0.05 was considered statistically significant.

## 3. Results

### 3.1. Cytokinesis-Block Micronucleus Assay (CBMN Assay)

**Figure 1:**
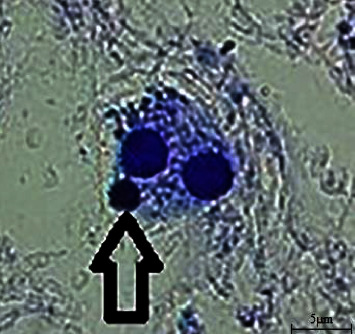
Giemsa stained, binucleated human lymphocyte cells; haloperidol-treated group at a concentration of 100 ng/ml (400X); the micronucleus is demonstrated with an arrow. Scale bar: 5 *µ*m.

**Figure 2:**
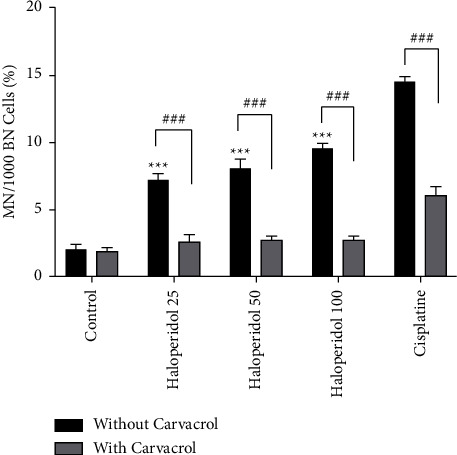
The protective effect of carvacrol on haloperidol-induced genotoxicity in MN frequency in human lymphocytes; values are presented as the mean ± SD; ^*∗∗∗*^significantly different compared to the control group without carvacrol (*P* < 0.001); ###significantly different in comparison with the haloperidol-treated group without carvacrol (*P* < 0.001).

CBMN was conducted to examine the probable genotoxic effect of haloperidol on lymphocytes as well as the protective effect of carvacrol. In order to assess the cytogenetic damage, the frequency of MN was measured in binucleated lymphocytes (Figure 1). As shown in Figure 2, the frequencies of MN increased significantly in samples treated with haloperidol in comparison with the control group (*P* < 0.001), whereas the frequency of MN significantly decreased in the groups treated with haloperidol and carvacrol (*P* < 0.001).

### 3.2. Alkaline Comet (SCGE) Assay

**Figure 3:**
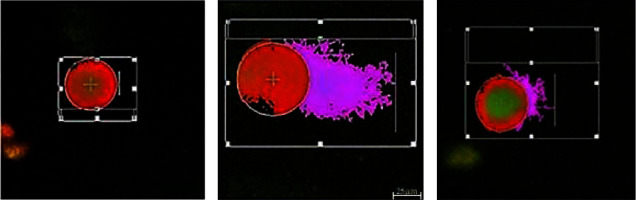
Demonstrating classes of comets: (a) no damage: control group (without the tail); (b) increased DNA damage: haloperidol-treated group; (c) decreased DNA damage: haloperidol + carvacrol treated group. Scale bar:25 *µ*m.

**Figure 4:**
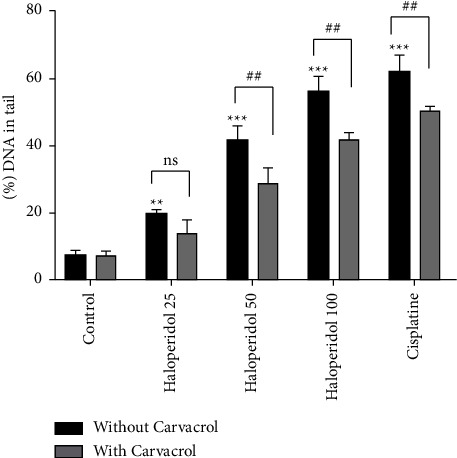
The percentage of DNA in the tail in human lymphocytes after treatment by carvacrol and haloperidol; values are presented as the mean ± SD; ^*∗∗*^significantly different compared to the control group without carvacrol (*P* < 0.01); ^*∗∗∗*^significantly different compared to the control group without carvacrol (*P* < 0.001); ##significantly different in comparison with the haloperidol-treated group without carvacrol (*P* < 0.01); ns: not significant.

**Figure 5:**
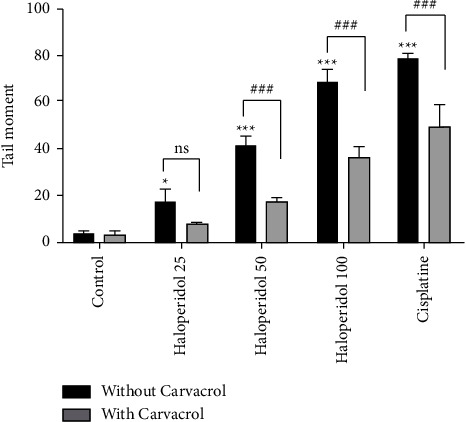
The changes in the tail moment in human lymphocytes after treatment by carvacrol and haloperidol; values are presented as the mean ± SD; ^*∗*^significantly different compared to the control group without carvacrol (*P* < 0.05); ^*∗∗∗*^significantly different from the control group without carvacrol (*P* < 0.001); ^###^significantly different from the haloperidol-treated group without carvacrol (*P* < 0.001); ns: not significant.

The alkaline comet (SCGE) assay is a sensitive method, which was performed in this study to evaluate haloperidol-induced DNA strand breaks and the effects of carvacrol on it.  Figure 3 shows the observed comets which were formed in each group. The results are demonstrated as the percentage of DNA in the tail (Figure 4) and the tail moment (Figure 5). As shown in both figures, the percentage of DNA in the tail and the tail moment increased significantly in the haloperidol group at all concentrations compared to the control group (*P* < 0.05). However, using carvacrol at haloperidol concentrations of 50 and 100 ng/ml decreased the percentage of DNA in the tail and tail moment (*P* < 0.05).

### 3.3. Measurement of Oxidative Stress Parameters

#### 3.3.1. Measurement of Lipid Peroxidation

**Figure 6:**
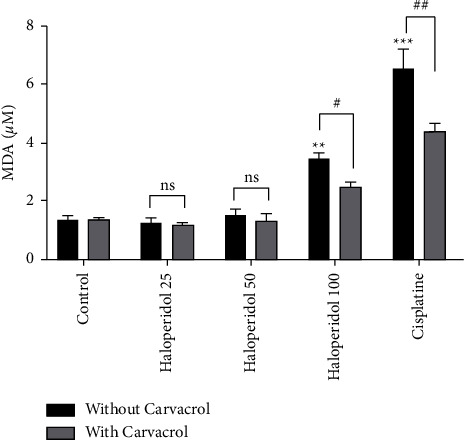
The protective effect of carvacrol on haloperidol-induced lipid peroxidation in human lymphocytes; MDA concentrations were evaluated as by-product of lipid peroxidation; values are presented as the mean ± SD; ^*∗∗*^significantly different compared to the control group without carvacrol (*P* < 0.01); ^*∗∗∗*^significantly different compared to the control group without carvacrol (*P* < 0.001); #significantly different in comparison with the haloperidol-treated group without carvacrol without carvacrol (*P* < 0.05); ##significantly different in comparison with the haloperidol-treated group without carvacrol (*P* < 0.01); ns: not significant.

The MDA level was assessed in this study as the final by-product of lipid peroxidation. The MDA levels significantly increased in the haloperidol treated groups at a concentration of 100 ng/ml, which is demonstrated in Figure 6 (*P* < 0.01). Whereas, the results indicated a statistically significant decrease when carvacrol was used with haloperidol at the concentration of 100 ng/ml (*P* < 0.05).

#### 3.3.2. Measurement of Glutathione Content

**Figure 7:**
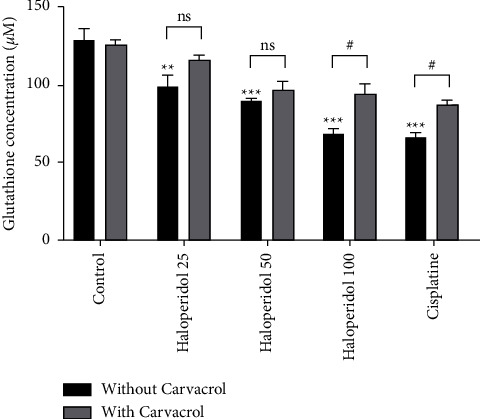
The protective effect of carvacrol on haloperidol-induced glutathione level in human lymphocytes; values are presented as the mean ± SD; ^*∗∗*^significantly different compared to the control group without carvacrol (*P* < 0.01); ^*∗∗∗*^significantly different compared to the control group without carvacrol (*P* < 0.001); #significantly different in comparison with the haloperidol-treated group without carvacrol (*P* < 0.05); ns: not significant.

Glutathione concentration was measured in this study as the other indicator of oxidative stress.  Figure 7 demonstrates significant decreases in the haloperidol group at all concentrations compared to the control group (*P* < 0.05), while the level of glutathione increased significantly when carvacrol was used with haloperidol at the concentration of 100 ng/ml (*P* < 0.05).

## 4. Discussion

Haloperidol is one of the most commonly prescribed antipsychotic drugs [39]. It has been reported in various studies that the induction of oxidative stress might be the principal mechanism correlated to haloperidol-induced adverse effects [40–42].

The main objective of this study was to provide further evidence on the probable potential of haloperidol in inducing genotoxicity through oxidative stress and the role of carvacrol in ameliorating it.

In order to demonstrate the genotoxic potential of haloperidol, we conducted the CBMN test and the alkaline comet assay, both of which can detect various genotoxic events in various cell lines [43, 44].

According to the results reported in various studies, it is presumed that the optimal therapeutic window of haloperidol is between 5 and 25 ng/ml. However, it is possible that the plasma concentration of a drug increases to 100 ng/ml or more as a consequence of drug accumulation in the case of drug overdose or chronic administration. So, we chose haloperidol concentrations close to these ranges [45–47]. Based on the results of this study, exposing human lymphocytes to different concentrations of haloperidol (25, 50, and 100 ng/ml) resulted in significant increases in the frequency of MN, DNA percentage in tail, and tail moment, indicating that haloperidol might have the potential to induce genotoxicity. This is consistent with the results reported in previous studies [36, 47–51]. In a similar study, the CBMN test and alkaline comet assay were conducted on human lymphocytes at lower concentrations of haloperidol (5, 10 and 20 ng/ml) compared to our study. The study found out that all mentioned concentrations of haloperidol increased DNA tail length significantly after 24 and 48 hours of exposure, in addition to a significant increase in the frequency of MN at 10 ng/ml. Interestingly, the results of this study indicated that haloperidol might cause genotoxicity in relatively clinical concentrations [36]. In another study, therapeutic concentrations (5–25 ng/ml) and higher concentrations (100 and 500 ng/ml) of haloperidol were evaluated using a chromosome aberration test. The results of the test demonstrated significant increases at 25, 100, and 500 ng/ml. Additionally, exposing cells to haloperidol decreased the mitotic index significantly at all concentrations, which provided further evidence that haloperidol may have harmful effects on the rate of mitosis through the inhibition of DNA synthesis [47]. The possible role of antipsychotics in changing the epigenetic status of the brain has been proposed and assessed in several studies [48–50]. The results of a recent study also revealed that haloperidol may potentiate the induction of DNA methylation in neuroblastoma cells. It was hypothesized that inducing DNA methylation is associated with haloperidol's strong blockade of dopamine D2 receptors [51]. The blockade of dopaminergic transmission by haloperidol leads to an increase in the turnover rate of dopamine, which will consequently result in the excessive production of reactive oxygen species and oxidative stress [52].

The key role of reactive oxygen species in the haloperidol-induced neurotoxicity was assessed in various studies. Based on their results, it was proposed that haloperidol may increase oxidative stress in cortical neurons of the brain, leading to a cascade of cellular events including an increase in the activity of the caspase-3 enzyme, which might result in cellular apoptosis, necrosis, and DNA fragmentation [42, 53, 54].

As regards oxidative stress, we found that haloperidol significantly reduced the level of cellular glutathione at all concentrations (*P* < 0.05), followed by a significant increase in lipid peroxidation at 100 ng/ml. This is in agreement with the findings of previous studies [41, 42, 55–63]. An early study on patients prescribed with haloperidol reported that a 2-week period of haloperidol administration significantly reduced the level of glutathione in the patients' cerebro-spinal fluid, in addition to a significant increase in MDA level [55]. Furthermore, in a study on male Sprague-Dawley rats that were chronically treated with haloperidol, it was revealed that haloperidol caused significant depletion in the levels of brain antioxidant defense enzymes such as superoxide dismutase (SOD) and glutathione peroxidase (GPx) [42]. In another study, male Wistar rats were under chronic treatment of haloperidol for 21 days. It was reported that treatment with haloperidol led to significant decreases in the levels of glutathione and the activity of antioxidant enzymes SOD and catalase, along with a significant increase in lipid peroxidation in the forebrain [56]. Exposing human plasma to haloperidol demonstrated significant increases in TBARS, which are accounted for as biomarkers of lipid peroxidation [57–59]. Moreover, the results of some studies indicated that haloperidol might cause hepatotoxicity and nephrotoxicity through the depletion of reduced glutathione and antioxidant enzyme activity as well as generating ROS in the kidney and liver [41, 60–63]. Additionally, the induction of oxidative stress by haloperidol was observed in a cell study. It was reported that the exposure of rat primary cortical neurons and hippocampal HT-22 cell lines to haloperidol initiated sequences of cellular changes that resulted in cell death. According to the findings of the study, haloperidol increased the generation of ROS and decreased the glutathione level, followed by increasing the intracellular Ca^2+^ level, which led to cell death [64].

The prominent role of antioxidants in reducing drug-induced cell death and cytotoxicity has been demonstrated in some studies [65–67]. In consonance with that, Behl et al. indicated that using vitamin E as a lipophilic antioxidant and free radical scavenger prevented DNA degradation and, therefore, necrotic cell death in random isolated cells treated with haloperidol. Based on the results of this study, it can be presumed that free radicals play the main role in haloperidol-induced necrotic cell death [68].

In recent years, the major role of natural antioxidants in preventing drug-induced side-effects correlated to oxidative stress has become a major area of focus [69]. Several studies have reported the alleviation of haloperidol-induced oxidative stress after treatment with natural antioxidant compounds [41, 42, 56, 59, 63, 70–72]. In our study, we also attempted to outline the potential of carvacrol as a natural antioxidant polyphenol compound. Carvacrol is a natural monoterpene, which has demonstrated anxiolytic and antidepressant properties in mice models [73, 74]. As a natural polyphenolic compound, carvacrol shares some common features with other compounds of the same family, including its ability to act as an antioxidant and scavenge free radicals, more specifically, reactive oxygen species. It is also assumed that monoterpenoids can have protective effects on the antioxidant defense system existing in the body [34, 75, 76]. Accordingly, the results of a TRAP/TAR assay (Total reactive antioxidant potential/Total antioxidant reactivity assay) revealed that carvacrol could scavenge peroxyl radicals [77]. The antioxidant properties of carvacrol are often attributed to the polyphenolic structure of this compound as well as its weak acid feature through which it can possibly react with free radicals and turn them into more stable radicals [78]. The results derived from various studies on drug-induced oxidative stress have also revealed that carvacrol improved glutathione levels significantly and restored the antioxidant defense enzymes [79–82]. Furthermore, carvacrol showed antilipid peroxidation ability by significantly decreasing markers such as MDA [83, 84]. We found that treating cells with carvacrol was effective in reducing haloperidol-induced oxidative stress and genotoxic events significantly. Our findings suggest that the concomitant administration of carvacrol as a supplement with haloperidol is a potential therapeutic strategy that might impose preventative impacts against haloperidol-induced adverse effects. However, further studies are required to be conducted.

## 5. Conclusion

In this study, we showed that haloperidol could mediate genotoxicity in human lymphocytes via the induction of oxidative stress. In addition, we observed that treating cells with carvacrol protected the cells against haloperidol-induced genotoxicity by reducing lipid peroxidation by-product MDA and increasing glutathione levels. Therefore, it can be presumed that oxidative stress plays an important role in haloperidol-mediated genotoxicity.

## Data Availability

The data used to support the findings of this study are available from the corresponding author upon reasonable request.
